# Impact of the COVID-19 Pandemic on Oncology Patients’ Mental Health and Treatment Plans

**DOI:** 10.3390/healthcare10050825

**Published:** 2022-04-29

**Authors:** Arwa Althumairi, Entesar Ahmed Al Askari, Reem S. AlOmar, Arwa Alumran

**Affiliations:** 1 Department of Health Information Management and Technology, College of Public Health, Imam Abdulrahman Bin Faisal University, Dammam 34212, Saudi Arabia; 2210500223@iau.edu.sa (E.A.A.A.); aalumran@iau.edu.sa (A.A.); 2 Department of Family and Community Medicine, College of Medicine, Imam Abdulrahman Bin Faisal University, Dammam 31451, Saudi Arabia; rsomar@iau.edu.sa

**Keywords:** COVID-19, mental health, psychology, oncology patients, Saudi Arabia, psycho-oncology

## Abstract

The COVID-19 pandemic has disrupted cancer care to a certain degree. There is objective evidence that COVID-19 outbreaks are causing substantial emotional distress among cancer patients regardless of their disease severity. This study aims to measure the levels of psychological distress, depression, and pandemic anxiety among cancer patients in Saudi Arabia during the outbreak of COVID-19 and their impact on patients’ cancer treatment plans. **Methods:** This was a cross-sectional study conducted among oncology patients in Saudi Arabia in November of 2020. The levels of stress, depression, and anxiety symptoms during the COVID-19 outbreak were measured using the Questionnaire for Depression and Anxiety (PHQ-4), and patients were classified as depressed/distressed if the total score was 6 and above and classified as not depressed/distressed if they scored less than 6. **Results:** Among the sampled population, anxiety symptoms and depression were detected in 61.5% and 70.2%, respectively. Statistical analyses revealed that feeling more isolated was significant for anxiety symptoms (*p* = 0.005), while patients who used institutions as a source of COVID-19 information had significant depression (*p* = 0.010) compared to patients who accessed information from other sources. In the binary regression model, feeling more isolated than before was 3.208 times more likely to be associated with anxiety symptoms (OR = 3.208; 95% CI = 1.391–7.396; *p* = 0.006), while those patients who had a support institution as a source of COVID-19 information were 4.2 times more likely to be associated with depression (OR = 4.200; 95% CI = 1.328–13.280; *p* = 0.015). **Conclusion:** The COVID-19 pandemic has added to the burden on cancer patients. The increased risk of anxiety symptoms and depression was clearly demonstrated in this study. Feeling isolated had a greater impact on anxiety symptoms, while obtaining COVID-19 information from a patient support institution negatively affected depression.

## 1. Introduction

The global outbreak of the novel coronavirus, also known as severe acute respiratory syndrome coronavirus 2 (SARS-CoV-2) or coronavirus disease 2019 (COVID-19), has become a major concern in health care and has caused unprecedented challenges in cancer care [1]. Cancer patients remain a distinctive population of patients encountering different challenges, including a high susceptibility to infectious diseases and a higher risk of COVID-19 outcomes [2]. The high susceptibility to COVID-19 in cancer patients is suggested to be a result of the immunosuppressive effects of their malignancies and the drugs, radiation, and surgical procedures used to treat them. In addition, cancer patients are at an increased risk of acquiring the infection in a medical centre as opposed to other patients [3].

The COVID-19 pandemic has disrupted cancer care, and this disruption includes the postponing of diagnoses and treatments. However, health care systems quickly restructured cancer services and changed their daily clinical practice, adapting measures and protocols for cancer care that enabled patients to continue receiving essential health services while minimizing exposure to COVID-19 [4,5].

These changes were made rapidly, and the newly revised guidelines were operationalized without carefully addressing the impact on the cancer patients’ psychological health, which is vital to increasing survival rates [2]. The few data reported on cancer patients confirm that they are subjected to substantial emotional stress related to their disease and are widely considered to be at a high risk for contracting and dying from COVID-19 [2]. For example, in a study that examined psychological distress among adolescent and young adult cancer patients from different countries, it was found that approximately 60% reported feeling more anxious than they did prior to COVID-19 [2]. However, this study concerned adolescents, and no study was found to assess the impact of COVID on patients in different age groups. The available literature that assessed the impact of COVID-19 on cancer patients was concerned with its impact on treatment and/or providing recommendations for adapting to living safely with the pandemic; however, none of the literature assessed the mental impact of COVID-19 on cancer patients of all ages [6,7,8]. Cancer survivors are known to have different psychological feelings in terms of anxiety and distress [9]. Changing treatment plans could impact the level of anxiety in cancer survivors, and this pandemic has had consequences in the changing of the administration of treatment [10]. Understanding the impact of COVID-19 on cancer patients can help in planning for care during future crises and in understanding the characteristics of cancer survivors.

In Saudi Arabia, several papers have examined the psychological distress of COVID-19 patients themselves, while others have targeted health care workers. To the best of our knowledge, none of these have studied the mental and psychological health of adult cancer patients. Examining this group of people will help in the development of recommendations to enhance daily clinical practice; and improve adapted measures and protocols for high-quality cancer care and patient safety. Therefore, this study aimed to measure the levels of psychological distress, depression, and pandemic anxiety symptoms among cancer patients in Saudi Arabia during the COVID-19 outbreak. The impact of the pandemic on survivors’ cancer treatment plans was also addressed.

## 2. Materials and Methods

### 2.1. Participants and Study Design

This cross-sectional study was performed in the Eastern Province of Saudi Arabia; adult cancer patients aged 18 years and above were included in the study. The study protocol was approved by the Institutional Review Board of Imam Abdulrahman Bin Faisal University. The expected sample size of the study was 392, which was calculated using a single population proportion formula with the assumption that 15% of cancer survivors would respond to the survey, and the study has a confidence level of 95%, a margin of error at 5%, and a response rate of 50. However, there were only 104 respondents to the study questionnaire that was distributed online through social media including WhatsApp, Twitter, and LinkedIn from 7 to 19 November 2020. This was during the middle of the pandemic, when social distancing was mandated and travelling in and out of the country was prohibited [11].

Table 1 shows the sociodemographic characteristics and the treatment plans of the cancer patients. The most common age group was 31–60 years old (55.8%), with nearly 60% being females and approximately three quarters (71.2%) being single. The categorizing of age group was to differentiate between young adults, older adults, and elderly adults. Additionally, 70.2% of cancer patients were living with their children. Nearly 60% were still undergoing their treatment process. The most common type of treatment was chemotherapy (41.3%), with 61.5% indicating that their treatment process had already been completed. The proportion of patients with chronic conditions was 56.7%, while the proportion of patients who used immunosuppressive drugs was 10.6%, and the proportion of patients who had mental health disorders was 6.7%.

### 2.2. Data Collection Tool

A questionnaire comprising two main parts was used. The first part included sociodemographic and health information such as age, sex, marital status, people with whom participants lived, type of treatment, chronic conditions, and immunosuppressant medication use. Second, psychological distress was measured with the Patient Health Questionnaire for Depression and Anxiety (PHQ-4), a 4-item measure of the frequency of 2 items for anxiety symptoms and 2 items for depressive symptoms on a 4-point Likert scale ranging from 0 (not at all) to 3 (nearly every day). The PHQ-4 scores indicate whether a patient’s symptoms are considered to be (0–2) normal, (3–5) mild, (6–8) moderate, or (9–12) severe. The participants were regrouped as having no anxiety/depression (normal to mild) or having anxiety/depression (moderate to severe). The reliability and validity of the brief PHQ-4 scale have been established in general and in clinical populations, and a total score of 6 or higher is indicative of clinically relevant distress [12]. The questionnaire was shared with one expert and two academics to review its validity and clarity. The questionnaire was also translated from English to Arabic and then to English again to ensure accuracy.

Accordingly, the questionnaire was distributed online and via social media to collect the information required for our study for two weeks from 7 to 19 November 2020.

The dependent variables were the depression and anxiety symptoms of cancer patients during the COVID-19 outbreak.

### 2.3. Statistical Analysis

Quantitative data are presented as the mean ± standard deviation (SD) and median (min-max) whenever appropriate. Qualitative data are presented using counts and proportions (%). For the comparisons between anxiety symptoms and patient characteristics as well as between depression levels and patient characteristics, chi squared tests were conducted. Binary regression analyses were conducted to determine the likelihood ratio of anxiety symptoms and depression among the significant factors drawn from the cross tabulations. A *p* value <0.05 (two sided) was used to indicate statistical significance. All data analyses were performed using the Statistical Package for Social Sciences, version 21 (SPSS, IBM Corp.: Armonk, NY, USA).

## 3. Results

Table 1 shows the association between anxiety symptoms and the treatment plans of cancer patients. Based on the results, age group in years, sex, marital status, living with others, treatment status, type of treatment, treatment disturbance, chronic conditions, immunosuppressant drugs, and mental health disorders were not significantly related to anxiety symptoms (all *p* > 0.05).

This study also found that age group in years, sex, marital status, living with others, treatment status, type of treatment, treatment disturbance, chronic conditions, immunosuppressant drugs, and mental health disorders had no significant relationship with depression (all *p* > 0.05) (Table 2).

Table 3 describes the impact of COVID-19 on cancer patients. Approximately 40% of patients reported that COVID-19 had an impact on their treatment protocol, follow-up, and appointments, with 26% indicating that it delayed their scheduled treatment. Furthermore, nearly half (49%) expressed that they were feeling more anxious and stressed at the height of the pandemic, while half also reported that they were feeling more isolated than before the COVID-19 pandemic. When asked about their satisfaction regarding the information they received about COVID-19, 65.4% expressed satisfaction; however, 91.3% stated that they still needed more information regarding the current pandemic.

Table 3 shows that feeling isolated showed a significant relationship with anxiety symptoms (X^2^ = 10.427; *p* = 0.005). Other statements regarding the impact of COVID-19 did not have a significant effect on anxiety symptoms (all *p* > 0.05).

Table 4 shows that the relationship between patients’ support institutions as the source of COVID-19 information and depression was statistically significant (X^2^ = 6.618; *p* = 0.010).

When conducting regression analysis, the risk of anxiety symptoms for those who felt that they were more isolated than before was three times higher than that for those who felt less isolated (OR = 3.208; 95% CI = 1.391–7.396; *p* = 0.006). The risk of depression for those who stated that patient support institutions were their source of COVID-19 information was four times higher (OR = 4.200; 95% CI = 1.328–13.280; *p* = 0.015) (Table 5).

## 4. Discussion

Emotional and psychological disorders greatly affect patients with a cancer diagnosis. The burden that is carried by the patients, including the treatment plan, can lead to emotional distress, including anxiety symptoms and depression. The purpose of the present study was to examine the levels of anxiety symptoms and depression among cancer patients in Saudi Arabia during the COVID-19 outbreak and their impact on the patients’ cancer treatment plans. The findings of this study revealed that the COVID-19 pandemic increased anxiety symptoms in cancer patients, with 61.5% exhibiting symptoms of anxiety. Consistently, several papers reported an increased risk of anxiety for cancer patients during the outbreak, varying from 19.1% in Singapore to 78% in the United States with many countries in between [13,14,15,16,17].

Furthermore, we learned that feeling isolated greatly affected anxiety symptoms. It can be further noted that the delay in treatment during the pandemic was minimal; however, an increase in perceived psychological distress was more obvious. Nearly half of the patients (49%) indicated that they felt more anxious and stressed during the pandemic, while feelings of isolation added to the burden on cancer patients. This is consistent with what was reported in previous literature, that cancer patients who were living alone had a higher risk of anxiety [13]. It was further added that patients who had a relative infected with the virus were more prone to suffer severe anxiety. In Singapore, anxiety was more prevalent among married patients, a finding which was also supported by a study in Poland [13,17]. However, in the current study, although those who had never been married were more anxious than those who had been married, the outcome did not show a significant impact on anxiety symptoms, which contradicted the previous findings.

In the UK, it was reported that women with breast cancer who had disruptions in their treatment plans during COVID-19 had high levels of anxiety and depression. This finding indicated that the restrictions due to COVID-19 lockdowns underlined the emotional and psychological susceptibility of women with primary breast cancer [18]. This scenario did not coincide with the current results, as cancer patients with mental disorders in this study were not greatly affected by anxiety.

Depression was another predictor of psychological distress among cancer patients during the COVID-19 outbreak. In this study, depression was detected in 70.2% of the study subjects. This is consistent with Miaskowski et al.’s work (2020), where they observed that 71.2% of oncology patients were considerably affected by depression during the outbreak [15]. Other papers reported a prevalence of depression ranging from 25.2% to 58% [13,14,16,19,20,21,22].

Interestingly, this paper revealed that COVID-19 information coming from the patient’s institution contributed to the increased risk of depression. This could be due to the amount and frequency of information received about COVID-19, especially by elderly patients and people with more comorbidities that would receive more information on COVID-19 and its related consequences [23]. Hence, the more information on restrictions is received, the more likely the receiver will be depressed.

The overall findings of this study add to the recent literature, which indicates that cancer patients are emotionally and mentally affected by the current pandemic [24,25]. Patients diagnosed with cancer are likely to suffer from the prolonged immunosuppressant effects of cancer along with its therapy [26]. Thus, the potential effects of delays and disruptions in treatment may lead to a considerable increase in the psychological distress associated with cancer [18].

## 5. Clinical Implications

The COVID-19 pandemic has added to the burden on cancer patients. The increased risk of anxiety and depression symptoms was clearly demonstrated in this study. Feeling isolated had a greater impact on anxiety levels, while obtaining COVID-19 information from patient support institutions negatively affected levels of depression. The potential effects of the COVID-19 pandemic can be detrimental to cancer patients. Effective measures should be undertaken to minimize psychological disorders and to provide mental health support. Further related research is needed to identify the extent of the effect of the COVID-19 pandemic on the cognitive, emotional, and psychological distress of cancer patients.

## 6. Study Limitations

The current study has some limitations. First, the limited sample size might have hindered in identifying the relations between some factors, such as sex. Additionally, the sample size did not sufficiently represent the target population, which might reduce the generalizability of the study. Second, the use of a cross-sectional survey-based study limited the generalizability of the study and limited the assessment of causation. However, this study did use a valid study tool to assess depression and anxiety symptoms. Finally, having baseline data on anxiety symptoms and depression levels before the pandemic and/or having a comparison group (patients with no cancer) would have provided a more accurate point of comparison. Nevertheless, a previous study supported the finding that people with cancer might experience an emotional impact from any discrepancy in their treatment plan; therefore, the study findings highlight the importance of monitoring treatment plans for patients with cancer.

## Figures and Tables

**Table 1:** Relationship between anxiety symptoms and cancer patient sociodemographic characteristics and treatment plans (*n* = 104).

Factor	Anxiety Symptoms				
	Yes	No	Total	X^2^	*p* Value
	N (%)	N (%)	N (%)		
	(*n* = 64)	(*n* = 40)	(*n* = 104)		
Age group					
19–30 years	8 (12.5%)	4 (10.0%)	12 (11.5%)	0.169	0.919
31–60 years	35 (54.7%)	23 (57.5%)	58 (55.8%)		
>60 years	21 (32.8%)	13 (32.5%)	34 (32.7%)		
Sex					
Male	24 (37.5%)	18 (45.0%)	42 (40.4%)	0.575	0.448
Female	40 (62.5%)	22 (55.0%)	62 (59.6%)		
Marital Status					
Never been married	47 (73.4%)	27 (67.5%)	74 (71.2%)	0.423	0.516
Been married	17 (26.6%)	13 (32.5%)	30 (28.8%)		
Living					
Without children	21 (32.8%)	10 (25.0%)	31 (29.8%)	0.718	0.397
With children	43 (67.2%)	30 (75.0%)	73 (70.2%)		
Treatment status					
Undergoing	38 (59.4%)	22 (55.0%)	60 (57.7%)	0.636	0.728
Last 6 months	9 (14.1%)	8 (20.0%)	17 (16.3%)		
More than 6 months	17 (26.6%)	10 (25.0%)	27 (26.0%)		
Type of treatment					
Chemotherapy	25 (39.1%)	18 (45.0%)	43 (41.3%)	4.91	0.178
Radiotherapy	7 (10.9%)	1 (2.5%)	8 (7.7%)		
Both	10 (15.6%)	11 (27.5%)	21 (20.2%)		
Others	22 (34.4%)	10 (25.0%)	32 (30.8%)		
Treatment disturbance					
Normal/completed	35 (54.7%)	29 (72.5%)	64 (61.5%)	3.856	0.145
Delayed	20 (31.3%)	9 (22.5%)	29 (27.9%)		
Interrupted	9 (14.1%)	2 (5.0%)	11 (10.6%)		
Chronic conditions					
Yes	33 (51.6%)	26 (65.0%)	59 (56.7%)	1.811	0.178
No	31 (48.4%)	14 (35.0%)	45 (43.3%)		
Immunosuppressant drugs					
Yes	9 (14.1%)	2 (5.0%)	11 (10.6%)	2.137	0.144
No/I don’t know	55 (85.9%)	38 (95.0%)	93 (90.4%)		
Mental health disorders					
Yes	4 (6.3%)	3 (7.5%)	7 (6.7%)	0.061	0.805
No/I don’t know	60 (93.8%)	37 (92.5%)	97 (93.3%)		

The *p* value was calculated using the chi-square test.

**Table 2:** Relationship between depression and cancer patient sociodemographic characteristics and treatment plans (*n* = 104).

Factor	Depressive Symptoms				
	Yes	No	Total	X^2^	*p* Value
	N (%)	N (%)	N (%)		
	(*n* = 73)	(*n* = 31)	(*n* = 104)		
Age group					
19–30 years	9 (12.3%)	3 (9.7%)	12 (11.5%)	0.177	0.915
31–60 years	40 (54.8%)	18 (58.1%)	58 (55.8%)		
>60 years	24 (32.9%)	10 (32.3%)	34 (32.7%)		
Sex					
Male	29 (39.7%)	13 (41.9%)	42 (40.4%)	0.044	0.834
Female	44 (60.3%)	18 (58.1%)	62 (59.6%)		
Marital Status					
Never been married	55 (75.3%)	19 (61.3%)	74 (71.2%)	2.093	0.148
Been married	18 (24.7%)	12 (38.7%)	30 (28.8%)		
Living					
Without children	19 (26.0%)	12 (38.7%)	31 (29.8%)	1.673	0.196
With children	54 (74.0%)	19 (61.3%)	73 (70.2%)		
Treatment status					
Undergoing	41 (56.2%)	19 (61.3%)	60 (57.7%)	5.418	0.067
Last 6 months	9 (12.3%)	8 (25.8%)	17 (16.3%)		
More than 6 months	23 (31.5%)	4 (12.9%)	27 (26.0%)		
Type of treatment					
Chemotherapy	29 (39.7%)	14 (45.2%)	43 (41.3%)	7.443	0.059
Radiotherapy	3 (4.1%)	5 (16.1%)	8 (7.7%)		
Both	14 (19.2%)	7 (22.6%)	21 (20.2%)		
Others	27 (37.0%)	5 (16.1%)	32 (30.8%)		
Treatment disturbance					
Normal/completed	48 (65.8%)	16 (51.6%)	64 (61.5%)	1.848	0.397
Delayed	18 (24.7%)	11 (35.5%)	29 (27.9%)		
Interrupted	7 (9.6%)	04 (12.9%)	11 (10.6%)		
Chronic conditions					
Yes	40 (54.8%)	19 (61.3%)	59 (56.7%)	0.374	0.541
No	33 (45.2%)	12 (38.7%)	45 (43.3%)		
Immunosuppressant drugs					
Yes	7 (9.6%)	4 (12.9%)	11 (10.6%)	0.253	0.615
No/I don’t know	66 (90.4%)	27 (87.1%)	93 (90.4%)		
Mental health disorders					
Yes	4 (5.5%)	3 (9.7%)	7 (6.7%)	0.611	0.434
No/I don’t know	69 (94.5%)	28 (90.3%)	97 (93.3%)		

The *p* value was calculated using the chi-square test.

**Table 3:** Impact of COVID-19 in relation to anxiety (*n* = 104).

Factor	Anxiety Symptoms				
	Yes	No	Total	X^2^	*p* Value
	N (%)	N (%)	N (%)		
	(*n* = 64)	(*n* = 40)	(*n* = 104)		
Impact on treatment protocols					
Yes	25 (39.1%)	16 (40.0%)	41 (39%)	1.095	0.578
Not yet, but I worry it will	11 (17.2%)	4 (10.0%)	15 (14%)		
No	28 (43.8%)	20 (50.0%)	48 (46%)		
Impact details					
No effect	39 (60.9%)	24 (60.0%)	63 (61%)	0.117	0.99
Delayed treatment	16 (25.0%)	11 (27.5%)	27 (26%)		
Cancelled appointment	2 (3.1%)	1 (2.5%)	3 (3%)		
Stopped treatment	7 (10.9%)	4 (10.0%)	11 (11%)		
Psychological well-being					
Better than before	7 (10.9%)	3 (07.5%)	10 (10%)	4.998	0.082
Same as before	21 (32.8%)	22 (55.0%)	43 (41%)		
More anxious and stressed than before	36 (56.3%)	15 (37.5%)	51 (49%)		
Feeling isolated					
Less isolated than before	4 (6.3%)	0	4 (4%)	10.427	0.005 **
Same as before	22 (34.4%)	26 (65.0%)	48 (46%)		
More isolated than before	38 (59.4%)	14 (35.0%)	52 (50%)		
Source of COVID-19 information *					
Medical professionals	37 (57.8%)	25 (62.5%)	62 (60%)	0.225	0.636
Social media and websites	54 (84.4%)	34 (85.0%)	88 (85%)	0.007	0.932
Patient support institution	21 (32.8%)	11 (27.5%)	32 (31%)	0.326	0.568
Relatives and friends	38 (59.4%)	19 (47.5%)	57 (55%)	1.401	0.236
Satisfaction about information received					
Satisfied	50 (78.1%)	35 (87.5%)	85 (82%)	1.449	0.229
Dissatisfied	14 (21.9%)	05 (12.5%)	19 (18%)		
Need more information					
Yes	59 (92.2%)	36 (90.0%)	45 (43%)	0.149	0.699
No	5 (7.8%)	04 (10.0%)	9 (9%)		

* Variable with multiple choice answer. The *p* value was calculated using the chi-square test. ** Significant at the *p* < 0.05 level.

**Table 4:** Impact of COVID-19 in relation to depression (*n* = 104).

Factor	Depression Symptoms				
	Yes	No	Total	X^2^	*p* Value
	N (%)	N (%)	N (%)		
	(*n* = 73)	(*n* = 31)	(*n* = 104)		
Impact on treatment protocols					
Yes	24 (32.9%)	17 (29.0%)	41 (39%)	5.556	0.062
Not yet, but I worry it will	10 (13.7%)	5 (16.1%)	15 (14%)		
No	24 (32.9%)	17 (54.8%)	48 (46%)		
Impact details					
No effect	49 (67.1%)	14 (45.2%)	63 (61%)	5.642	0.13
Delayed treatment	17 (23.3%)	10 (32.3%)	27 (26%)		
Cancelled appointment	1 (1.4%)	2 (6.5%)	3 (3%)		
Stopped treatment	6 (8.2%)	5 (16.1%)	11 (11%)		
Psychological well-being					
Better than before	8 (11.0%)	2 (6.5%)	10 (10%)	4.237	0.12
Same as before	34 (46.6%)	9 (29.0%)	43 (41%)		
More anxious and stressed than before	31 (42.5%)	20 (64.5%)	51 (49%)		
Feeling isolated					
Less isolated than before	3 (4.1%)	1 (3.2%)	4 (4%)	1.149	0.563
Same as before	36 (49.3%)	12 (38.7%)	48 (46%)		
More isolated than before	34 (46.6%)	18 (58.1%)	52 (50%)		
Source of COVID-19 information *					
Medical professionals	47 (64.4%)	15 (48.4%)	62 (60%)	2.313	0.128
Social media and websites	64 (87.7%)	24 (77.4%)	88 (85%)	1.757	0.185
Patient support institution	28 (38.4%)	4 (12.9%)	32 (31%)	6.618	0.010 **
Relatives and friends	43 (58.9%)	14 (45.2%)	57 (55%)	1.659	0.198
Satisfaction about information received					
Satisfied	61 (83.6%)	24 (77.4%)	85 (82%)	0.55	0.458
Dissatisfied	12 (16.4%)	07 (22.6%)	19 (18%)		
Need more information					
Yes	67 (91.8%)	28 (90.3%)	45 (43%)	0.059	0.809
No	6 (8.2%)	3 (9.7%)	9 (9%)		

* Variable with multiple choice answer. The *p* value was calculated using the chi-square test. ** Significant at the *p* < 0.05 level.

**Table 5:** Regression analysis to determine the factors independently associated with anxiety symptoms and depression (*n* = 104).

Anxiety Symptoms	Odds Ratio	95% CI	*p* Value
Feeling isolated			
Same as before	Ref		
Less isolated than before	0.000	0.000	0.999
More isolated than before	3.208	1.391–7.396	0.006 **
**Depression**			
Patient support institution as source of COVID-19 information			
No	Ref		
Yes	4.200	1.328–13.280	0.015 **

CI—confidence interval. ** Significant at the *p* < 0.05 level.

## Data Availability

The data presented in this study are available on request from the corresponding author. The data are not publicly available due to patient approval statement.
